# Recombinant Lipidated HPV E7 Induces a Th-1-Biased Immune Response and Protective Immunity against Cervical Cancer in a Mouse Model

**DOI:** 10.1371/journal.pone.0040970

**Published:** 2012-07-18

**Authors:** Chiung-Yi Huang, Jeremy J. W. Chen, Kuan-Yin Shen, Li-Sheng Chang, Yi-Chen Yeh, I-Hua Chen, Pele Chong, Shih-Jen Liu, Chih-Hsiang Leng

**Affiliations:** 1 National Institute of Infectious Disease and Vaccinology, National Health Research Institutes, Zhunan Town, Miaoli, Taiwan, Republic of China; 2 Institute of Molecular Biology, National Chung Hsing University, Taichung, Taiwan, Republic of China; 3 Graduate Institute of Immunology, China Medical University, Taichung, Taiwan, Republic of China; University of Bergen, Norway

## Abstract

The E7 oncoprotein of human papillomavirus (HPV) is an ideal target for developing immunotherapeutic strategies against HPV-associated tumors. However, because protein-based immunogens alone are poor elicitors of the cytotoxic T-lymphocyte (CTL) responses, they have been difficult to exploit for therapeutic purposes. In this study, we report that a recombinant lipoprotein consisting of inactive E7 (E7m) biologically linked to a bacterial lipid moiety (rlipo-E7m) induces the maturation of mouse bone marrow-derived dendritic cells through toll-like receptor 2 (TLR2), skews the immune responses toward the Th1 responses and induces E7-specific CTL responses. We further studied the ability of rlipo-E7m to provide protection against a TC-1 tumor cell challenge in an animal model. Mice prophylactically immunized with two 10-µg doses of rlipo-E7m were found to be free of TC-1 tumor growth. Experiments in a therapeutic immunization model showed that the tumor volume in mice receiving a single dose of rlipo-E7m was less than 0.01 cm^3^ on day 40, whereas the tumor volume in mice treated with rE7m was 2.28±1.21 cm^3^. The tumor volume of the entire control group was over 3 cm^3^. In addition, we demonstrated that the CD8+ T cells play a major role in anti-tumor immunity when administration of rlipo-E7m. These results demonstrate that rlipo-E7m could be a promising candidate for treating HPV-associated tumors.

## Introduction

Human papillomavirus (HPV) infections may account for the development of several cancers; in particular, HPV infection can cause cervical cancer. Cervical cancer is the second leading cause of cancer deaths in women worldwide [1]. Therefore, novel therapies are urgently needed to control cervical cancer mortality. To date, HPV E6 and E7 oncoproteins have been identified as the major targets for development of therapeutic vaccines against HPV-associated tumors. Moreover, as intracellular (nuclear) proteins, the E6 and E7 gene products may be hidden from the humoral immune response. Attention has thus focused on the generation of a vaccine capable of inducing or stimulating a cellular immune response to HPV E6 and E7 oncoproteins [2]. Several approaches have been taken to develop HPV therapeutic vaccines, including live vector-, peptide/protein-, nucleic acid- and whole cell-based approaches. These studies have shown the importance of T-cell responses in protecting against tumors in human and animal models [3]. Of these approaches, synthetic peptide and protein-based approaches are the most attractive candidates; however, in general, peptide and protein alone are non-immunogenic. Without additional adjuvant, it is difficult to induce strong, effective immune responses characterized by the production of Th1 cytokines and viral oncoprotein-specific cytotoxic T-lymphocytes (CTLs) [3].

TLR agonists are naturally immuno-stimulatory inducers of innate immunity. TLR2 is expressed on many different cell types, including dendritic cells, macrophages, and lymphocytes. Therefore, the TLR2 agonists such as bacterial lipoproteins are promising candidates to be used as a new generation adjuvant for immunotherapy.

We have recently established a platform for high-yield production of recombinant lipoproteins [4]. The lipid moiety of the lipo-immunogen is identical to that of bacterial lipoproteins, which are recognized as danger signals by the immune system. Thus, both innate and adaptive immune responses can be induced by lipoproteins [5], [6]. The lipid structure of the recombinant lipoprotein is different from that of synthetic tri-acylated lipopeptide [7]. This distinction makes recombinant lipoprotein elicited different immune responses from synthetic lipopeptide by inducing different levels of biological cytokines and chemokines [8]. We have previously shown that virus-neutralizing antibody responses elicited by lipid form of an immunogen were stronger than those elicited by a non-lipid form of an immunogen [4], [9]. In this report, we demonstrate that the difficulties of using proteins alone to induce CTL responses could be overcome by using the lipo-immunogen strategy. A lipid form of the E7 mutant (rlipo-E7m) and a non-lipid form of rE7m from HPV type 16 (HPV16) were produced, and the ability of these immunogens to activate mouse bone marrow-derived DCs (BM-DCs) was assessed. The immune responses of B and T cells were also investigated. Lastly, we examined whether the immune responses induced by rlipo-E7m were able to protect mice against tumor challenge. These studies not only provide information about rlipo-E7m as a promising approach for the development of a therapeutic vaccine against HPV-associated tumors but also present a strategy for the development of successful immunotherapies using protein-based candidates.

## Results

### Production of Recombinant Immunogens

**Figure 1:**
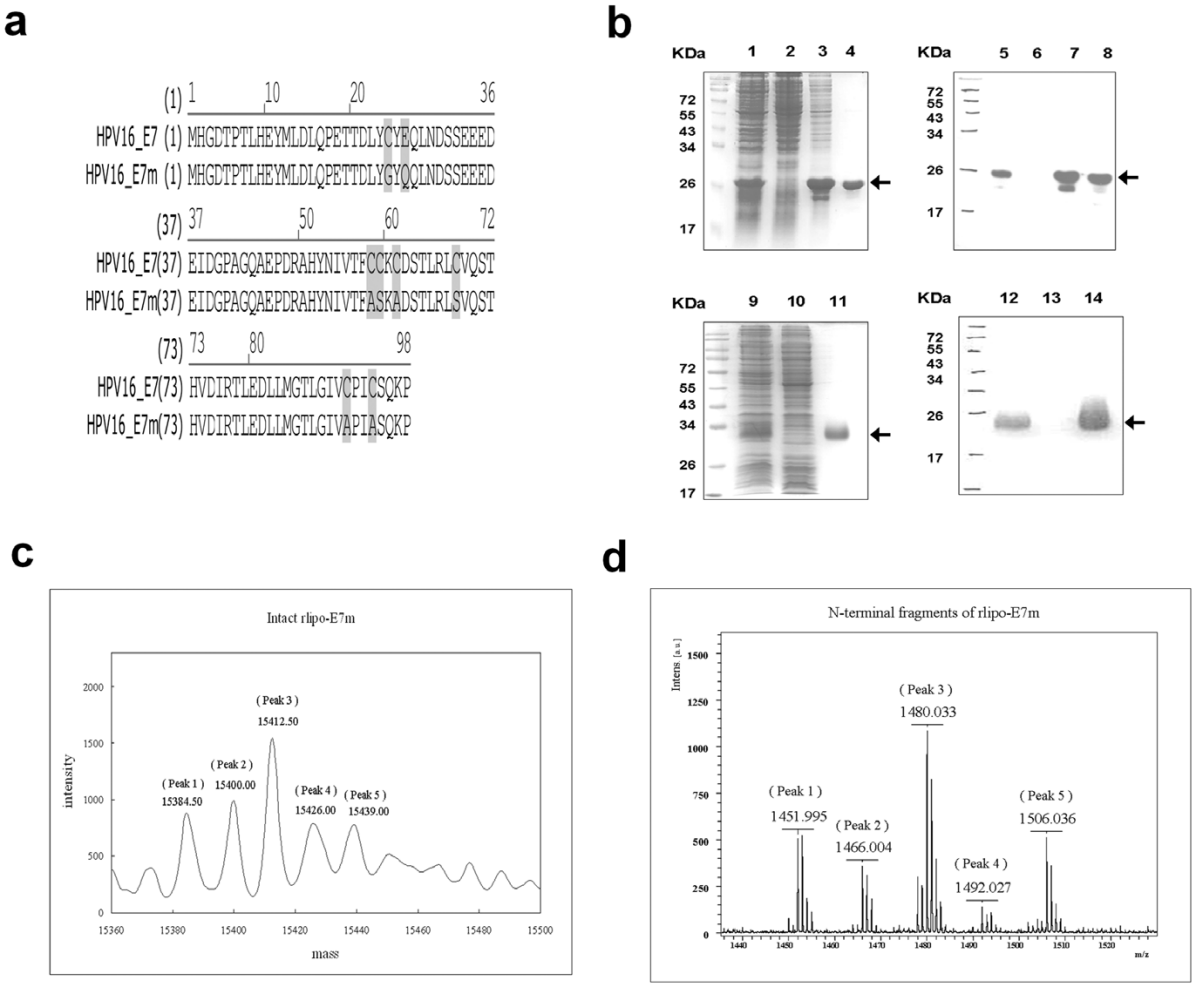
Construction, production and identification of rE7m and rlipo-E7m. (**a**) The alignment of the amino acid sequence of wild-type HPV16 E7 (Accession number: NP_041326) and the inactive E7 (E7m). Every mutant site of E7m is highlighted. The DNA sequence of the E7m gene was derived using codon usage of *E. coli* and fully synthesized using the assembly PCR method. The PCR product was cloned into the pET22b vector for rE7m expression and into the modified pET22b vector with a lipid signal peptide for rlipo-E7m expression. The recombinant proteins contained an additional HHHHHH sequence (HisTag) at the C-terminus and were expressed under the control of the T7 promoter. (**b**) The rlipo-E7m and rE7m protein purification process was monitored using 15% reducing SDS-PAGE followed stained by Coomassie Blue staining and immunoblotting using anti-HisTag antibodies. The recombinant rlipo-E7m was expressed in *E. coli* strains C43 (DE3). Lane 1, rE7m expression after IPTG induction; lane 2, protein expression in the absence of IPTG induction; lane 3, extracted fraction of rE7m; lane 4, purified recombinant rE7m. Lanes 5–8 show immunoblotting to monitor the rE7m purification process, and the samples in these lanes are the same as those in lanes 1–4, respectively. Lane 9, rlipo-E7m expression after IPTG induction; lane 10, protein expression in the absence of IPTG induction; lane 11, purified rlipo-E7m. Lanes 12–14 show immunoblotting to monitor the rlipo-E7m purification process, and the samples in these lanes are the same as those in lanes 9–11, respectively. The non-lipidated form of rE7m was expressed in *E. coli* BL21 (DE3) star strain. The arrows indicate the electrophoretic positions of rE7m or rlipo-E7m in the gels or blots. (**c**) Intact rlipo-E7m was analyzed by LC/MS. LC/MS analysis revealed the presence of five major post-translational modifications of rlipo-E7m. Molecular masses (in daltons) were determined using a maximum entropy algorithm. The five major peaks displayed masses of 15384.5, 15400, 15412.5, 15426 and 15439. (**d**) N-terminal rlipo-E7m fragments were obtained and identified after digestion of rlipo-E7m with trypsin. The digested sample was analyzed on a Waters® MALDI micro MX™ mass spectrometer. The MALDI-TOF MS spectra revealed the existence of five peaks with m/z values of 1452, 1466, and 1480, 1492 and 1506.

The recombinant E7m (rE7m), which was engineered to contain a hexahistidine tag (HisTag) at its C-terminus, was produced to compare the effects of rE7m to the lipidated target. rE7m was purified using immobilized metal affinity chromatography (IMAC) column (**Figure 1b** **, lanes 1–4**). rE7m was detected with anti-HisTag antibodies (**Figure 1b** **, lanes 5–8**). We previously identified a fusion sequence (D1) that can be fused with a non-lipidated immunogen to achieve high expression levels of the recombinant lipo-immunogen [4]. Using this strategy, the E7m gene was fused to D1 at its N-terminus and engineered a HisTag at its C-terminus. This recombinant lipidated E7m (rlipo-E7m) was purified using IMAC column **(** **Figure 1b** **, lanes 9–11**). rlipo-E7m was detected with anti-HisTag antibodies (**Figure 1b** **, lanes 12–14**). After the lipopolysaccharide (LPS) was removed (less than 0.003 EU/µg), purified rlipo-E7m and rE7m were comparatively analyzed for their immunogenicity and efficacy in animal models.

### Identification and Characterization of Purified Immunogens

rlipo-E7m and rE7m were digested with trypsin to assess their peptide mass fingerprinting (PMF). The results confirmed that the major peaks in the mass spectra were derived from rlipo-E7m and rE7m (data not shown). To identify the lipid moiety of rlipo-E7m, direct sample infusion ESI-MS of intact rlipo-E7m was performed on a Q-TOF instrument. After processing the data, five major peaks with masses of 15384.5, 15400, 15412.5, 15426, and 15439 Da were identified (**Figure 1c**). Based on our previous studies, we considered the existence of these major peaks as the signature of lipid modifications of the lipoproteins [4], [7].

We then measured the exact mass of N-terminal fragments of rlipo-E7m. Five peaks with m/z values of 1452, 1466, 1480, 1492, and 1506 were identified (**Figure 1d**). Purification of the typtic lipo-fragments for mass analysis revealed two more peaks that have not previously been identified. In order to verify that the lipid modification of each peak is in the N-terminal cysteinyl residue, N-terminal fragments of rlipo-E7m were subjected to MALDI-TOF-TOF analysis to determine the sequence of each peak. The sequences of these five peaks were the same, and the identified sequence from y5 to y1 was “SQEAK” (data not shown). We confirmed that the peaks of rlipo-E7m were associated with the lipidated cysteine residue and verified that rlipo-E7m contains a bacterial lipid moiety at its N-terminus.

### rlipo-E7m Activates Bone Marrow-derived Dendritic Cells Through TLR2

**Figure 2:**
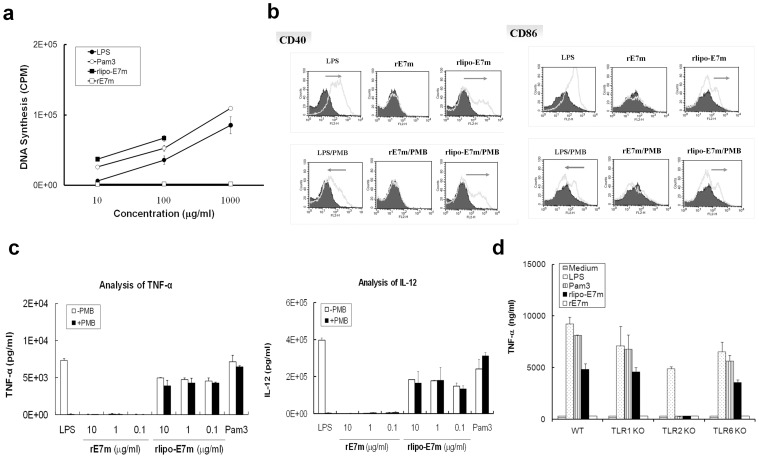
Bone marrow-derived dendritic cells (BM-DCs) are activated by rlipo-E7m through TLR2. (**a**) Splenocytes were isolated from wild-type mice and plated at a density of 2×10^5^ cells/well in 96-well plates. The cells were incubated with various concentrations of LPS (0–1000 µg/ml), Pam3 (0–1000 µg/ml), rlipo-E7m (0–100 µg/ml) or rE7m (0–1000 µg/ml) for 48 hrs. During the final 24 hrs, 1 µCi of [^3^H]-thymidine was added to each well to measure DNA synthesis. The data are represented as the mean ± SD of triplicate samples. (**b**) BM-DCs from wild-type mice were cultured in medium supplemented with LPS (0.01 µg/ml), rE7m (10 µg/ml) or rlipo-D1E3 (10 µg/ml) in the presence or absence of polymyxin B (20 µg/ml). After a 24-hr incubation, the expression of the dendritic cell surface markers CD40 and CD86 was analyzed using flow cytometry. Experiments were performed in triplicate and the mean fluorescence intensity (MFI) of cells cultured in medium alone was defined as 100%. (**c**) For the cytokine secretion studies, BM-DCs were cultured in medium supplemented with LPS (0.01 µg/ml), Pam3 (0.15 µg/ml), rE7m (0.1–10 µg/ml) or rlipo-E7m (0.1–10 µg/ml) in the presence or absence of polymyxin B (20 µg/ml). After a 24-hr incubation, the supernatants were harvested and analyzed for TNF-α and IL-12 (p40) production by ELISA. The data are presented as the mean±SD from three independent experiments. (**d**) BM-DCs from wild-type, TLR1-knockout (TLR1-KO), TLR2-KO or TLR6-KO mice were cultured either in medium alone or in medium supplemented with LPS (0.01 µg/ml), Pam3 (0.15 µg/ml), rE7m (10 µg/ml), or rlipo-E7m (10 µg/ml). After a 24-hr incubation, the supernatants were harvested and analyzed for TNF-α production by ELISA. The data are represented as the mean±SD of triplicate samples.

To assess the function of rlipo-E7m, we first examined the lymphocyte proliferation response to the recombinant immunogens and to control agonists. The LPS (a TLR4 agonist) and the synthetic lipopeptide palmitoyl-3-Cys-Ser-(Lys)4 (Pam3, a TLR2 agonist) induced the proliferation of splenocytes in a dose-dependent manner. rlipo-E7m was found to stimulate the splenocytes at concentrations of 10 nM and 100 nM [rlipo-E7m aggregated at 1000 nM in Phosphate-buffered saline (PBS)]. In contrast, rE7m failed to induce splenocyte proliferation. These results demonstrate that the lipid moiety of rlipo-E7m is able to activate immune cells (**Figure 2a**).

We then used BM-DCs as a model to study the immuno-stimulatory properties of rlipo-E7m. Polymyxin B was added to monitor any interference caused by residual LPS in functional assay. rlipo-E7m up-regulated the expression of the surface markers such as CD40 and CD86 on BM-DCs, whereas rE7m had no effect (**Figure 2b**). Similar results were obtained in cytokine secretion studies. The secretion of TNF-αand IL-12p40 was induced by rlipo-E7m but not by rE7m group (**Figure 2c**). Polymyxin B inhibited LPS-mediated stimulation of BM-DCs, but it had no significant effect on the stimulatory properties of rlipo-E7m. These results indicate that the activation of BM-DCs by rlipo-E7m was not due to residual LPS and that the immuno-stimulatory activity of rlipo-E7m was linked to its lipid moiety.

Because the lipid structure of rlipo-E7m is an agonist for TLR1/TLR2 and/or TLR2/TLR6, the next examined which of these TLRs is essential for the immuno-stimulatory activity of rlipo-E7m. To address this question, BM-DCs from wild-typed, TLR1-, TLR2- and TLR6-knockout (KO) mice were used. rlipo-E7m and Pam3 induced TNF-α secretion in BM-DCs from TLR1-KO and TLR6-KO mice but failed to stimulate BM-DCs from TLR2-KO mice, indicating that TLR2 is necessary for the immuno-stimulatory activity of rlipo-E7m (**Figure 2d**). These data demonstrate that the immunogen fused with bacterial lipid moiety activated the maturation of BM-DCs through TLR2.

### Immunization with rlipo-E7m Enhances Antigen-specific Antibodies and Generates a Th1-biased Response

**Figure 3:**
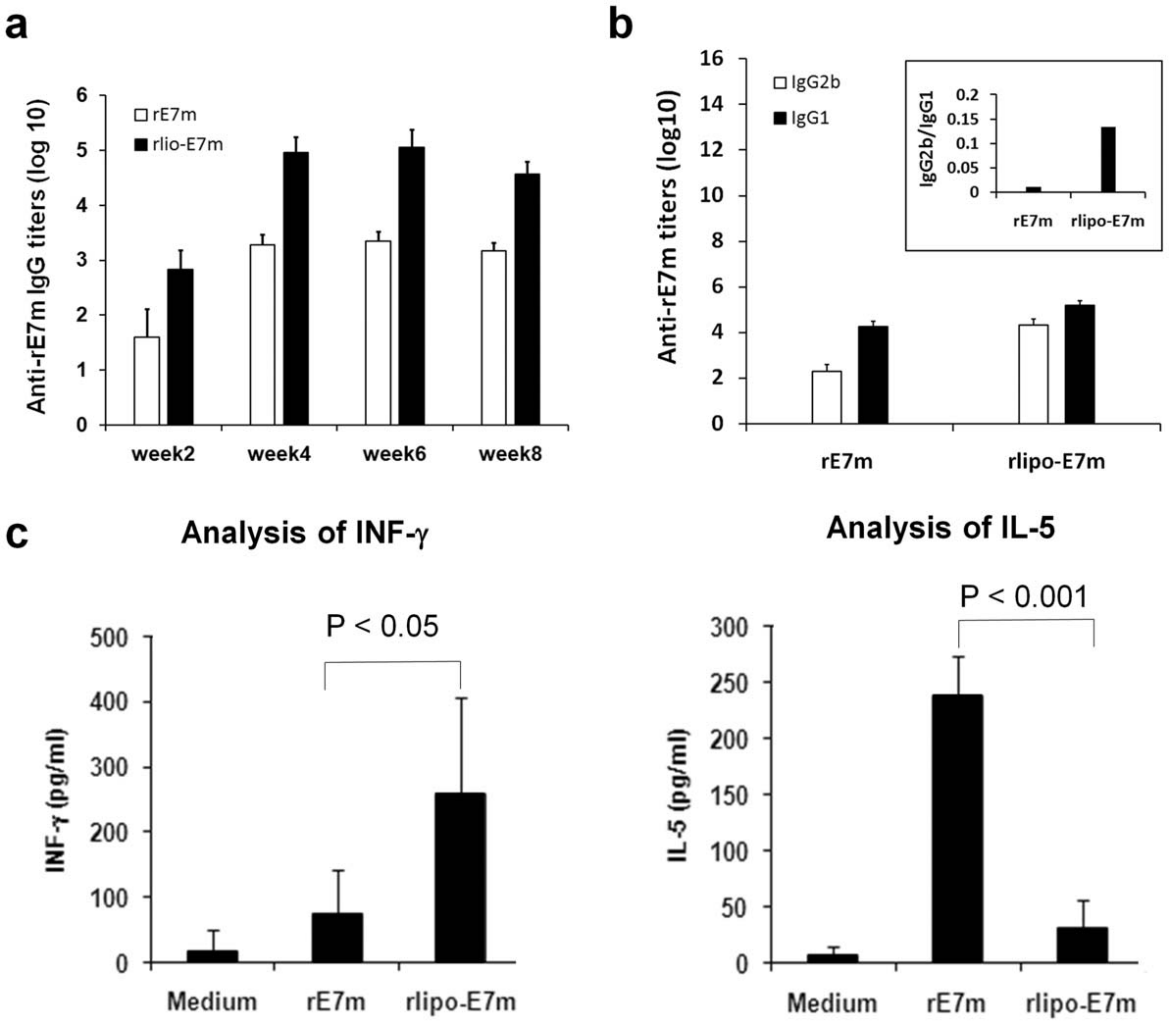
Enhancement of anti-E7m IgG antibody titers and induction of a Th1-biased immune response after administration of rlipo-E7m. (**a**) C57BL/6 mice (n = 5) were immunized twice by subcutaneous injection of 30 µg of rE7m in PBS or of 30 µg of rlipo-E7m in PBS at two-week intervals. Sera were collected, and IgG or (**b**) IgG2b/IgG1 responses against rE7m were evaluated by ELISA. The IgG2b/IgG1 ratios in both groups are shown in the insert. (**c**) Mice were injected subcutaneously of 30 µg of rE7m or rlipoE7m twice at two-week intervals. Seven days after the second immunization, splenocytes were isolated and stimulated with rE7m (10 µg/ml) for 4–5 days. The supernatants were collected, and the levels of IFN-γor IL-5 were measured by ELISA. Data are expressed as means+SD of samples (n = 5).

To evaluate the intrinsic adjuvant properties of rlipo-E7m *in* *vivo*, we analyzed the magnitude of the antigen-specific antibody response in mice immunized with either rE7m or rlipo-E7m (**Figure 3a**). The antibody titers elicited by rlipo-E7m were 100-fold higher than those elicited by rE7m at week 4, 6 and 8. Subsequently, to analyze the antibody isotypes elicited upon immunization with rE7m or rlipo-E7m, the induced levels of IgG1 and IgG2b were measured. The IgG1 levels were comparable in both rE7m- and rlipo-E7m-immunized mice; however, the IgG2b levels in the rlipo-E7m-immunzed mice were higher than those in the rE7m-immunized mice (**Figure 3b**). This phenomenon can be clearly observed by comparing the IgG2b/IgG1 ratios in these mice (**Figure 3b** **, insert**).

Furthermore, we examined the secretion of INF-γ and IL-5 in splenocytes of mice immunized with rE7m or rlipo-E7m. The level of INF-γinduced upon rlipo-E7m immunization was 4-fold higher than that induced upon rE7m immunization (**Figure 3c**); however, the rE7m immunization induced a 7-fold higher secretion of IL-5 by splenocytes than did the rlipo-E7m immunization (**Figure 3c**). Together, the results of the T cell and B cell assays suggest that fusing an immunogen to a bacterial lipid moiety results in a Th1-based immune response induced by the lipidated immunogen.

### Immunization with rlipo-E7m Induces E7-specific T Cell Responses

**Figure 4:**
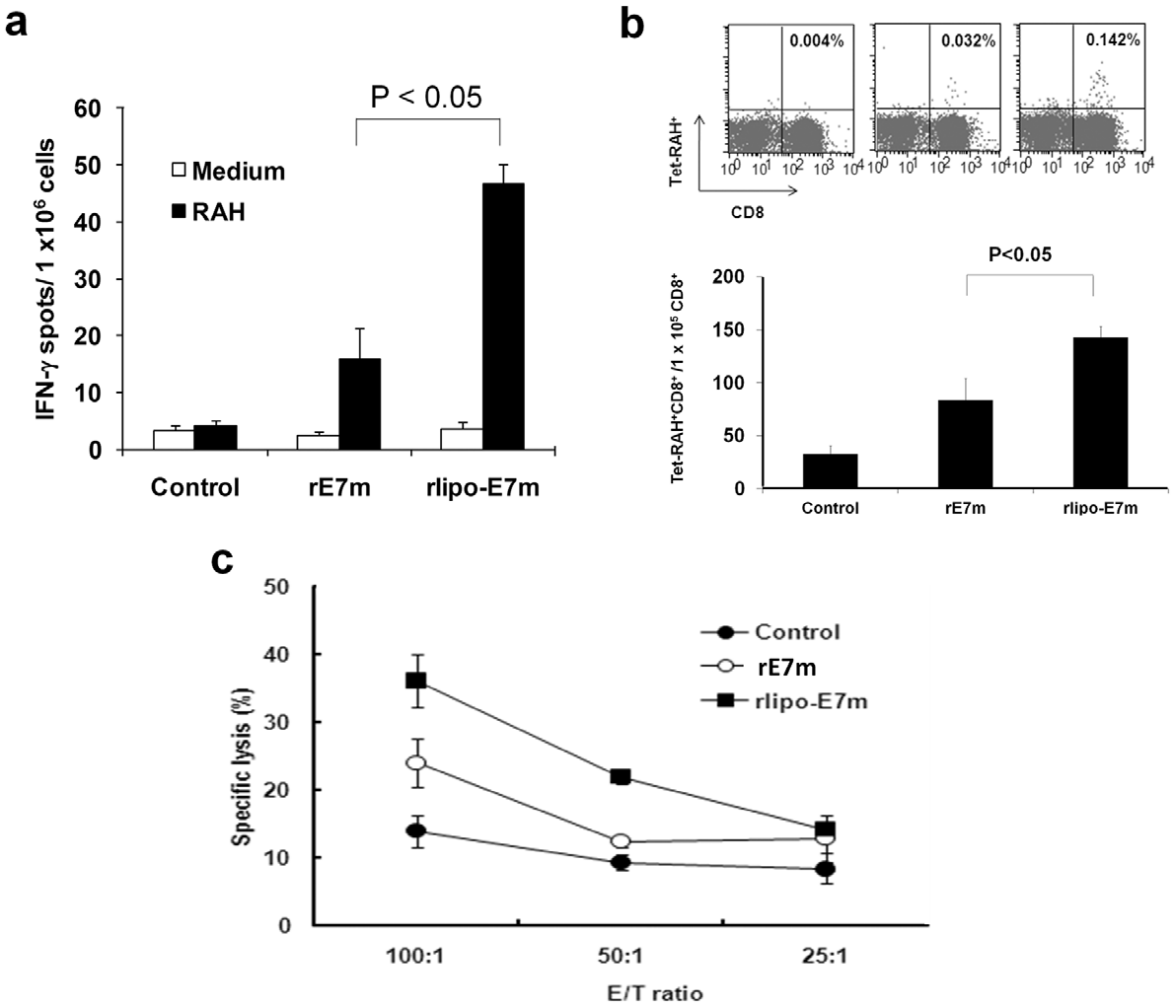
Immunization with rlipoE7m induces higher levels of E7-specific cytotoxic T lymphocyte activity. (**a**) Splenocytes (2×10^5^ cells/well) from immunized mice were incubated with or without 10 µg/ml of RAHYNIVTF (RAH) peptide for 48 hrs in an anti-IFN-γ-coated 96-well ELISPOT plate. The IFN-γ-secreting spots were measured using an ELISPOT reader. Data are expressed as means+SD of six animals per group. (**b**) The RAH-specific CD8+ T cells were detected by tetramer staining and analyzed by flow cytometry. The percentage of tetramer-positive cells among the CD8+ T cells is indicated within each panel. The numbers of TetraRAH-tetramer +/CD8+ cells in total CD8 cells (1×10^5^) from three experiments were showed. Bar graphs indicating the mean percentage of RAH tetramer+/CD8+ cells among the CD8+ cells. Data are expressed as the mean + SD. *P<0.05. (**c**) To assess the cytotoxic effect of rlipo-E7m immunization on tumor cells, a standard chromium-release assay was performed. Isolated splenocytes (effector cells) were stimulated with RAH (10 µg/ml) for 7 days in the presence of rIL-2 (10 unit/ml). The target cells (5×10^3^/well) were incubated with different ratio of effector cells (1∶25, 1∶50, 1∶100). Specific lysis (%) was calculated as follows: 100 x (Sample releasecpm – Spontaneous releasecpm)/(Maximum releasecpm – Spontaneous releasecpm).

We then investigated whether rlipo-E7m immunization was able to induce E7-specific CTL responses. C57BL/6 mice were subcutaneously immunized on day 0 and 7 with rlipo-E7m, rE7m or a PBS control. The splenocytes of the immunized mice were stimulated with peptide RAHYNIVTF (RAH, E7_49-57_) and the number of E7-specific IFN-γ-secreting cells was determined using ELISPOT assay (see Materials and Methods). Immunization with rlipo-E7m induced a higher number of INF-γsecreting cells than that did immunization with rE7m (**Figure 4a**). Additionally, staining with an RAH/H-2D^b^ R-Phycoerythrin (PE)-conjugated tetramer demonstrated a dramatic increase in E7-specific CD8+ T cells after immunization with rlipo-E7m. The number of E7-specific CD8+ T cells induced by rlipo-E7m immunization was more than 4 times that induced by rE7m immunization (**Figure 4b**). To test T cell killing activity after rlipo-E7m or rE7m immunization, RAH peptide-pulsed T2 cells were used as target cells in a standard 4-hr chromium release assay. rlipo-E7m immunization induced a high level of E7-specific CTL activity. In contrast, rE7m immunization only induced a low level of E7-specific CTL activity (**Figure 4c**). These results clearly demonstrate that the HPV E7-specific CTL responses were induced by rlipo-E7m vaccination.

### Evaluation of the Therapeutic and Prophylactic Efficacy of rlipo-E7m

**Figure 5:**
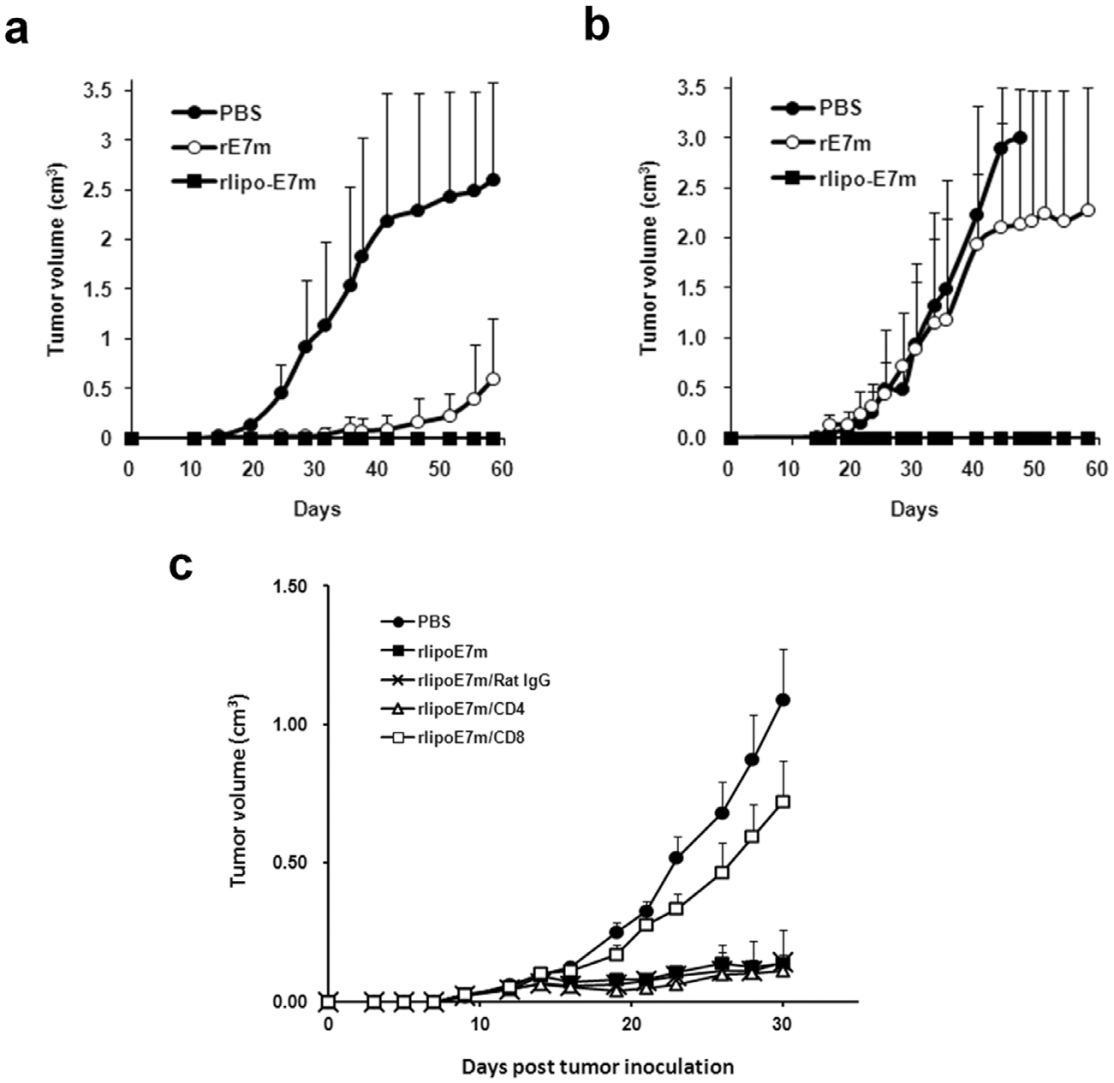
Immunization with rlipo-E7m induces a strong anti-tumor effect. (**a**) Mice were immunized twice by subcutaneous injection of rE7m/rlipo-E7m (10 µg) or PBS at one-week intervals. Seven days after the final immunization, mice were subcutaneously injected with 2×10^5^ TC-1 cells in a total volume of 200 µl subcutaneously. (**b**) Mice were subcutaneously inoculated with TC-1 cells (2×10^5^/mouse). After 7 days, tumor-bearing mice (5–10 mice per group) were injected once with rE7m/rlipo/rE7m (30 µg/mouse) or PBS. The tumor volume was shown as length x width x width/2 (mm^3^). Data are expressed as means+SEM. (**c**) Three groups (rlipo-E7m/CD4, rlipo-E7m/CD8 and rlipo-E7m/Rat IgG) of mice were injected of anti-CD4, anti-CD8 and control antibodies, respectively, one day before the injection of TC-1 cells. These groups and two additional groups (rlipo-E7m and PBS) were injected once with rlipo-E7m (10 µg/mouse) or PBS seven days after inoculated with TC-1 cells (2×10^5^/mouse). The tumor volume was shown as length x width x width/2 (mm^3^). Data are expressed as means+SEM.

In the prophylactic model, mice were immunized twice with PBS, rE7m or rlipo-E7m at two weeks interval, and the TC-1 cells were subcutaneously injected at 7 days after last immunization. On day 58 after challenge, the tumors remained barely measurable in rlipo-E7m-immunized mice. In contrast, the average tumor volume in mice injected with PBS was larger than 2.5 cm^3^, and the average tumor volume was 0.59±0.62 cm^3^ in mice vaccinated with rE7m (**Figure 5a**). To assess the therapeutic model, C57BL/6 mice were subcutaneously injected with TC-1 cells at dorsum. Seven days later, these mice received 30 µg of either rlipo-E7m or rE7m or received only PBS via subcutaneous injection of the abdomen. On day 58 post-challenge, the tumors were scarcely detectable in mice receiving rlipo-E7m. In contrast, the average tumor volume in mice injected with PBS was over 3 cm^3^, and the average tumor volume in mice immunized with rE7m was greater than 2.0 cm^3^ (**Figure 5b**). These results demonstrate that both the prophylactic and therapeutic efficacies of recombinant immunogens are dramatically increased when they are delivered in a lipidated form.

Five groups of C57BL/6 mice were used to further identify which subset of T cells contributes to the anti-tumor immunity. Three groups were injected of anti-CD4 (rlipo-E7m/CD4), anti-CD8 (rlipo-E7m/CD8) and control antibodies (rlipo-E7m/Rat IgG) one day before the injection of TC-1 cells. Seven days later, these mice received 10 µg of rlipo-E7m. Treatments of PBS and 10 µg of rlipo-E7m were served as negative and positive controls, respectively. On day 30 after tumor inoculation, the average tumor volume of PBS and rlipo-E7m groups were 1.1 cm^3^ and 0.14 cm^3^, respectively. No significant differences among the groups of rlipo-E7m, rlipo-E7m/CD4 and rlipo-E7m/Rat IgG. On the contrary, the protective effect was abolished when CD8+ cells were depleted in rlipo-E7m/CD8 group (p<0.005, **Figure 5c**). This result evidently demonstrated that administration of rlipo-E7m could generate CD8+ cells mediated anti-tumor effect.

## Discussion

We have produced a lipidated immunogen, rlipo-E7m, which consists of the inactivated E7 protein of HPV16 biologically fused to a bacterial lipid moiety. Our results demonstrate that the rlipo-E7m alone induces Th1 immune responses, E7-specific CTL responses, and tumor-protective immunity in a mouse tumor model.

The challenge of developing protein-based immunotherapies against tumors is that the protein-based immunogens themselves have low immunogenicity and do not readily elicit CTL responses. Formulating the immunogen with adjuvants is the most common approach to developing effective vaccines and overcoming the limitation of protein-based immunotherapies. We have extended this idea by covalently linking an immunogen to an immunopotentiator, creating a lipoprotein-based vaccine with intrinsic adjuvant activity.

Early studies demonstrated the powerful adjuvant activity of bacterial lipoprotein, synthetic lipids to the peptide antigens and lipid-tailed glyco-peptides [10], [11], [12]. For example, the recombinant lipidated OspA could induce protective immunity correlated with the development of antibodies. This Lyme disease vaccine (LYMErixTM) was licensed by US FDA in 1998 [13], [14]. Another recombinant lipoprotein approach is based on the outer membrane lipoprotein I (OprI) from Pseudomonas aeruginosa. OprI-based vaccine formulation using classical swine fever (CSF) E2 and NS3 promoted DC activation in the pig, resulting in enhanced T cell responses along with humoral immune defenses [15]. The OprI has also been fused with a tuberculosis vaccine candidate, mycolyl-transferase antigen 85A (Ag85A). Subcutaneous boosting with this fusion protein in the absence of adjuvant increased significantly the Ag85A-specific humoral immune responses [16]. MALP-2 (macrophage-activating lipopeptide-2) has shown encouraging anti-tumoral activity in some models [17], [18]. Oldford and coworkers demonstrated that Pam3CSK4 inhibited tumor growth in a mast cell and mast cell-derived IL-6-dependent manner [19]. Later a three-component GLP vaccine by Ingale and coworkers (a three palmitic acid Pam3CSK4 moiety, a CD4+ and a B-cell epitope) showed induction of strong tumor-specific IgG responses [20]. Recently, Renaudet and coworkers showed a four-component HER-GLP vaccine construct (one CD4+ T-cell epitope, one OVA CD8+ T-cell epitope, one carbohydrate B-cell epitope and a built-in palmitic acid adjuvant) inhibited the tumor growth in HER-GLP immunized mice [21].

Adjuvant use of lipopeptides elicits both Th1 and Th2 cytokines depending on the model antigen used in immunizations. For instance, totally synthetic lipopeptides including palmitoyl-lipidated peptide and cholesterol-lipidated peptide were found that could trigger a Th1-dependent protective immunity [22]. In contrast, the diacylated lipopeptide FSL-1 induces TLR2-mediated Th2 responses [23]. However, the immune responses can be skewed by lipoproteins and lipopeptides toward the Th1 responses were still observed many times. A synthetic pam3 lipopeptide of OspA induce Th1 phenotype development in αβ T-cell receptor transgenic mice [5]. Bettahi and coworkers found that the lipopeptide elicited a polarized Th1 immune response [24]. Imanishi and coworkers showed that the Pam3-CysSK4 and MALP-2 as a specific activator of Th1 cell function and imply the involvement in Th1-mediated responses [25].

We have demonstrated that a lipo-immunogen could successfully enhance the generation of neutralizing antibodies against dengue virus [4], [7]. Here, we used rlipo-E7m to demonstrate that lipoprotein immunogens can induce tumor-specific CTL responses. Importantly, this advanced approach provides a rational design for cancer immunotherapies for the future. We believe that this lipo-immunogen-approach can be developed into a new low-cost and effective treatment for HPV-associated cancers. Moreover, the lipid structure itself is a TLR2 agonist [26] and can go through the unfolding and refolding process during preparation without affecting its function. This advantage allows recombinant lipo-immunogens to retain more flexibility and allows for the robust production of lipidated immunogens. Although the approval of lipidated OspA was questioned about the arthritogenic responses caused by the molecular mimicry of OspA, no safety issues were raised regarding the lipid moiety of recombinant lipo-immunogens [27]. In our platform technology, we have optimized the upstream and downstream processes for production of recombinant lipoprotein, rAg473 of *Neisseria meningitides* group B, and have characterized the rAg473 using the optimized processes [28]. The pre-clinical toxicology and animal studies of rAg473 have been completed and we already provided the safety data to Taiwan Food and Drug Administration for Investigational New Drug (IND) application.

In order to induce CTL responses, protein-based immunogens normally must be formulated with adjuvants (review [3] [29]). For instance, the saponin-based adjuvant ISCOMATRIX [30] and the liposome-polycationic-DNA (LPD) adjuvant [31] have been shown to improve CTL responses of HPV protein-based vaccines. HPV-16 E7 has been fused to a fragment of *Haemophilus influenzae* protein D formulated in an adjuvant system containing Monophosphoryl Lipid A and QS-21 saponin adjuvant [32]. Recently, Nventa Biopharmaceuticals (purchased by Akela Pharma) reported that the efficacy of HSPE7 was further enhanced by the adjuvant Po1y-ICLC, and this finding served as the foundation for the phase 1 clinica1 trials [3]. Although recent advances in the adjuvant field have provided several potent and useful adjuvant candidates, developing efficient and safe adjuvants for human use remains a challenge [33]. Our approach avoids the hurdle of using adjuvant, suggesting that it may provide a new method to pursue the development of therapeutic vaccines.

In conclusion, we have demonstrated that biologically fusing a tumor antigen with a bacterial lipid moiety rendered this antigen capable of stimulating the BM-DCs through TLR2, inducing a Th1 immune response, inducing antigen-specific CTL responses, and generating tumor-protective immunity in a mouse tumor model. rlipo-E7m may thus be a promising therapeutic vaccine candidate against HPV-associated tumors. Moreover, *E. coli* has been used as a stable production system for biologics, and this lipidation technology can be easily applied to other tumor-associate antigens with few limitations. This strategy may provide a method for the development of successful immunotherapies using protein-based candidates and will hopefully yield the safety and effective vaccines for human use.

## Materials and Methods

### Chemicals

All chemicals were purchased from Sigma (St. Louis, MO) and Merck (Darmstadt, Germany). Restriction enzymes and ligase were purchased from New England Biolabs, Inc. (Beverly, MA). Primers used for cloning were purchased from Mission Biotech, Inc. (Taipei, Taiwan). Trypsin and the matrix used for mass spectrometry analysis were purchased from Promega Co. (Madison, WI).

### Cell Lines and Medium

TC-1, a mouse epithelial cell line transformed with the oncogenes Ras, HPV16 E6 and E7, was a kind gift from Dr. T-C Wu (Johns Hopkins University). TC-1 cells were cultured in DMEM (GIBCO-BRL, Grand Island, NY) supplemented with 10% heat-inactivated fetal bovine serum (HyClone, Logan, Utah), penicillin (100 units/ml) and streptomycin (100 µg/ml). Complete RPMI-10 medium contained RPMI-1640 supplemented with 10% (v/v) heat-inactivated fetal calf serum, 25 mM HEPES, 4 mM L-glutamine, 100 units/ml penicillin, 100 µg/ml streptomycin sulfate and 50 µM β-mercaptoethanol.

### Rationale for the Design of Inactive HPV E7 (E7m)

The HPV E7 oncoprotein contains three conserved regions: domains CR1, CR2, and CR3 [34]. The N-terminal domains of the E7 protein, CR1 and CR2, contribute significantly to cell transformation, and these two conserved regions are highly homology with the E1A proteins of adenovirus and also with related sequences of large tumor antigens in simian vacuolating virus 40 [35]. We have mutated the CR2 conserved LXCXE motif and the CR3 conserved Cys-X-X-Cys regions of HPV16 E7 to block the binding of E7 to the pRb pocket region [36], [37] and to abolish the binding of the pRb-E2F complex [34], [37], respectively. In addition to mutating LYCYE to LYGYQ in the CR2 region, all cysteine residues of E7 were mutated to alanine or serine to inactive the function of the CR3 region and to avoid the non-homogeneous protein generated by diverse pairing of disulfide bond [38]. The resulting protein, referred to as the inactive-E7 immunogen (E7m) (**Figure 1a**), still contains most identified CTL epitopes [39].

### Cloning and Expression of Recombinant Proteins

The E7m gene was obtained using an assembly PCR method with overlapping primers [40]. The product of the assembly PCR was then amplified by conventional PCR. To generate an expressing plasmid for recombinant E7m, the following primers were used: forward primer, 5′-GGAATTCCATATGCACGGCGATACCCCGACCCTGC -3′ (Nde I site, underlined); reverse primer, 5′- AGAGCCGCTCGAGCGGTTTCTGGCTCGCAATCGG -3′ (Xho I site, underlined). The PCR product was cloned into the expression vector pET-22b(+) (Novagen, Madison, WI), using Nde I and Xho I sites to produce the pE7m plasmid. As a result, the C-terminus of the recombinant protein contained an additional hexahistidine tag (HisTag). The *E. coli* strain BL21Star (DE3) (Invitrogen, Carlsbad, CA) was transformed with the expression plasmid, pE7m for protein expression. The transformed cells were cultured at 37°C overnight, and protein expression was induced by adding 1 mM IPTG for 3 h. To generate the expression plasmid for the lipidated immunogen, we modified the plasmid, pD1E3, which can be transformed into the CD43(DE3) strain for expression of recombinant lipoprotein, rlipo-D1E3 [4]. The primers used for this step were as follows: forward primer, 5′- CGCGGATCCATGCACGGCGATACCCCGACCCT-3′ (Bam HI site, underlined); reverse primer, 5′- AGAGCCGCTCGAGCGGTTTCTGGCTCGCAATCGG-3′ (Xho I site, underlined). The PCR product was cloned into pD1E3 using Bam HI and Xho I sites to produce the pD1E7m plasmid. As a result, the C-terminus of the recombinant protein contained an additional hexahistidine tag (HisTag). The *E. coli* strain C43(DE3) (Invitrogen, Carlsbad, CA) was transformed with the expression plasmid pD1E7m for lipo-protein expression. The transformed cells were cultured at 37°C overnight, and protein expression was induced by adding 1 mM IPTG at 12°C for 3 days.

### Purification of Recombinant Proteins

The un-lipidated recombinant E7m (rE7m) was expressed in *E. coli* BL21(DE3) star strain. After lysing the harvested cells with BugBuster master mix (Novagen, Madison, WI), the cell lysate was clarified by centrifugation (80,000 x *g* for 40 min). The pellet was resuspended in extraction buffer [2 M Urea/50 mM Tris (PH8.0)] and was homogenized with a Dounce homogenizer. The mixture was then clarified by centrifugation (80,000 x *g* for 40 min). The supernatant was incubated with 5 ml Chelating Sepharose (GE Healthcare, Waukesha, WI) coupled with copper and rocked at cold room overnight. The resin was loaded into a column (1.6 cm i.d. x 2.5 cm) and washed with the extraction buffer. rE7m was then eluted with extraction buffer containing 100 mM imidazole. A polymyxin B agarose column (Pierce, Rockford, IL) was used to remove lipopolysaccharide (LPS) and to exchange the buffer with phosphate buffer saline (PBS). The amount of residual LPS in rE7m preparations was determined by the Limulus amebocyte lysate (LAL) assay (Associates of Cape Cod Inc., East Falmouth, MA). LPS levels were reduced to less than 0.003 EU/µg.

The recombinant lipidated E7m (rlipo-E7m) was purified by disrupting the harvested cells in a French Press (Constant Systems, Daventry, UK) at 27 Kpsi in homogenization buffer [50mM Tris (PH8.0)]. The cell lysate was clarified by centrifugation (80,000 x *g* for 40 min). rlipo-E7m was solubilized from the pellet using solubilization buffer [1% Triton X-100; 50 mM Tris (PH8.9)]. The supernatant was incubated with 5 ml of Ni-NTA resin (Qiagen, San Diego, CA) overnight and loaded into a column (1.6 cm i.d. x 2.5 cm). The column was washed with the solubilization buffer, and rlipo-E7m was eluted with extraction buffer containing 100 mM imidazole. After being dialyzed against solubilization buffer, the purified rlipo-E7m was bound to Chelating Sepharose resin coupled with copper for removing the LPS. After extensively washing with solubilization buffer, the solubilization buffer was exchanged with PBS. LPS levels were found to be below 0.003 EU/µg.

### Analysis of Purified Recombinant Proteins

The purified rlipo-E7m was analyzed by SDS-PAGE, immunoblotting, and N-terminal amino acid sequencing. To measure the molecular weight of intact rlipo-E7m, intact rlipo-E7m was directly infused into a Q-TOF instrument (Waters, SYNAPT™ High Definition Mass Spectrometry™) using a manual acquisition mode. The molecular mass was calculated using 30 iterations of the maximum entropy algorithm MaxEnt1 (Waters). We modified our previous procedure to identify the N-terminal fragments of rlipo-E7m. Briefly, after digestion, the reaction mixture was further polished using Ziptip (Millipore, Massachusetts). One microliter of the polished tryptic fragments was mixed with 1 µl of a saturated solution of α-ciano-4-hydroxycinnamic acid in acetonitrile/0.1% trifluoroacetic acid (1∶3, vol/vol). One microliter of the mixture was placed on the target plate of an Autoflex III MALDI-TOF-TOF instrument (Bruker Daltonics, Bremen, Germany) for analysis.

### Activation of BM-DCs

BM-DCs derived from wild-type, TLR1-KO, TLR2-KO or TLR6-KO mice were assessed as previously described [4]. Briefly, mouse bone marrow cells were cultured at a density of 2×10^5^ cells/ml in petri dishes containing 10 ml of complete RPMI-1640 medium with 200 unit/ml (20 ng/ml) recombinant mouse GM-CSF. On day 3, an additional 10 ml of complete RPMI medium containing 20 ng/ml GM-CSF was added. On day 6, the cells were collected from each dish, washed and counted. BM-DCs (1×10^6^ cells/ml) were stimulated with LPS (10, 100, and 1000 ng/ml) or the indicated concentrations of rE7m, rlipo-E7m or Pam3CSK4 for 24 hrs. Cell surface markers (CD40 and CD86) of BM-DCs were analyzed using flowcytometry (FACSCalibur, BD bioscience, San Jose, CA). The production of cytokines (TNF-α and IL-12p40) by BM-DCs was determined using ELISA kits (R&D Systems, Minneapolis, MN).

### ELISPOT Assay

IFN-γ-secreting cells were analyzed using an IFN-γ ELISPOT assay as previously described [8]. Briefly, splenocytes (5×10^5^/well) were added to anti-IFN-γ-coated plates and cultured in presence of 10 µg/ml of the indicated peptides in a final volume of 200 µl RPMI-10. After incubation, the cells were removed by washing the plates with 0.05% (w/v) Tween 20 in PBS. A 50-µl aliquot containing 10 µg/ml of biotinylated anti-IFN-γ antibody (clone R46A2, eBioscience San Diego, CA) was added to each well, and samples were incubated for 2 hrs. The spots were developed using 3-amine-9-ethyl carbazole (Sigma, St. Louis, MO) and counted by ELISPOT reader (Cellular Technology Ltd., Shaker Heights, OH).

### Splenocytes Proliferation Assay

Splenocytes from C57BL/6 mice were plated at a density of 2×10^5^/well in 96-well plates and stimulated with LPS (10, 100, and 1000 ng/ml) or the indicated concentrations of rE7m, rlipo-E7m or Pam3CSK4 for a total of 48 hrs at 37°C in the presence of 5% CO_2_ in a humidified incubator. In the final 24 hrs of culture, 1 µCi of [^3^H]-thymidine (5 µCi/mmol; Amersham, Arlington Heights. IL) was added to each well, and cells were harvested using a FilterMate (Packard, Meriden, CT) automatic cell harvester. The incorporated radioactivity was determined using a TopCount microplate scintillation counter (Packard, Meriden, CT). LPS was included in the assay as a positive control. All results are presented as the mean cpm ± standard deviation (SD).

### Analysis of Antibodiese

C57BL/6 or TLR2-KO mice were immunized twice by subcutaneous injection of 30 µg of rE7m in PBS or of 30 µg of rlipo-E7m in PBS at two-week intervals. The anti-E7m antibody titers were determined using an ELISA assay. The total IgG, IgG1 and IgG2b titers were detected with horseradish peroxidase-conjugated goat anti-mouse IgG (ICN Biomedicals Inc., Aurora, OH), rabbit anti-mouse IgG1 (Zymed Laboratories Inc., San Francisco, CA) and rabbit anti-mouse IgG2b (Zymed Laboratories Inc., San Francisco, CA) respectively. After the addition of 3, 3′, 5, 5′-tetramethylbenzidine (TMB), the absorbance was measured with an ELISA reader at 450 nm.

### T-cell Assay

Female C57BL/6 mice were immunized twice by subcutaneous injection of 30 µg of rE7m or rlipo-E7m at 14-day intervals. On day 7 after the second immunization, mice were sacrificed, and splenocytes were harvested and stimulated with rE7m (10 µg/ml) for 4 days. Supernatants were collected, and cytokine (IL-5 and IFN-γ) production was analyzed using an ELISA kits (R&D Systems, Minneapolis, MN). To analyze E7-specific CD8+ T cells, splenocytes were stimulated with 10 µg/ml of H-2D^b^-restricted E7-derived peptide (RAH: RAHYNIVTF) overnight. The E7-specifc CD8+ T cells were stained with (PE)-conjugated H-2D^b^/RAH tetramer and anti-CD8-FITC. The percentage of H-2Db/RAH+ CD8+ T cells was determined by flow cytometry (FACSCalibur, BD Bioscience, San Jose, CA).

For cytotoxic T lymphocytes assay, erythrocyte-depleted splenocytes (1×10^6^ cells/ml) were cultured *in vitro* with E7-derived peptides (10 µg/ml) and unit/ml recombinant human IL-2 (Pepro tech, Rocky Hill, NJ) in 24-well plates for 5 days. On day 5, the TC-1 cells (5×10^5^) were pulsed with selected peptides and labeled with 100 µCi of ^51^Cr (Na251CrO4, PerkinElmer, MA) at 37°C for 1 hr; these were the target cells. The peptide-stimulated splenocytes were harvested and mixed with labeled target cells at splenocyte:labeled target cell ratios of 100∶1, 33∶1 and 1∶1. After incubation for 4 hrs, the supernatants were harvested to measure the radioactivity using a gamma counter. The percentage of specific lysis was calculated as follows: 100 x [(experimental release – spontaneous release)/(maximal release – spontaneous release)].

### Animal Studies

Six- to twelve-week-old female C57BL/6 mice were obtained from the National Laboratory Animal Breeding and Research Center (Taipei, Taiwan). All animals were housed at the Animal Center of National Health Research Institutes (NHRI) and maintained in accordance with the institutional animal care protocol. All of the animal studies were approved by the animal committee of the NHRI for this study (approval ID: NHRI-IACUC-097077-A). For the tumor protection experiment, C57BL/6 mice (six per group) were immunized with 10 µg of rE7m or rlipo-E7m. Mice were boosted once with the same regimen used for the first vaccination. Two weeks after the last vaccination, mice were challenged with 2×10^5^ TC-1 tumor cells/mouse by subcutaneous injection in the right leg and monitored twice a week by inspection and palpation. In the therapeutic model, mice were first injected with 2×10^5^ TC-1 tumor cells. On the seventh day after the tumor challenge, 30 µg of rE7m or rlipo-E7m were administratered to C57BL/6 mice (six per group). Tumor diameters were measured in two orthogonal dimensions using a caliper two or three times per week. Tumor volumes were calculated from the measurements according to the following formula: (length x width^2^)/2.

### Depleting Mice of CD4+ or CD8+ T Lymphocytes

T cell-depletion experiments have been described previously [41]. Briefly, groups of mice were treated intraperitoneally (i.p.) with either 0.5 mg of rat anti-CD4 antibodies (clone GK1.5, eBioscience), or rat anti-CD8 antibody (clone 53-6.72, eBioscience) to deplete CD4+ and CD8+ T lymphocytes, respectively. As a control, 0.5 mg of rat IgG (Sigma, Deisenhofen, Germany) was administered in experiments. Efficacy of depletion was greater than 90% as determined by flow cytometry using different clones of fluorescence conjugated anti-CD4 or anti-CD8 antibodies. Antibodies were injected before the inoculation of 2×10^5^ TC-1 tumor cells. On the seventh day after the tumor inoculation, 10 µg of rE7m or rlipo-E7m were administered to C57BL/6 mice (six per group).
